# Photodynamic Therapy (PDT) in Oncology

**DOI:** 10.3390/cancers12113341

**Published:** 2020-11-12

**Authors:** Ángeles Juarranz, Yolanda Gilaberte, Salvador González

**Affiliations:** 1 Department of Biology, Faculty of Sciences, Universidad Autónoma de Madrid, 28049 Madrid, Spain; 2 Department of Dermatology, University Hospital Miguel Servet, IIS Aragón, 50009 Zaragoza, Spain; ygilaberte@gmail.com; 3 Department of Medicine and Medical Specialties, Alcalá de Henares University, 28805 Madrid, Spain; salvagonrod@gmail.com

The issue is focused on Photodynamic Therapy (PDT), which is a minimally invasive therapeutic modality approved for treatment of several types of cancer and non-oncological disorders. PDT is being used in dermatology for the treatment of non-melanoma skin cancers (basal cell carcinoma, BCC, and Bowen disease, BD) and precancerous lesions (actinic keratosis, AKs), among other tumors such as: head and neck cancer and bladder and gynaecological neoplasms. PDT can also be used for cancer diagnosis by means of theragnosis (a term arising from the combination of diagnostic tests and therapy), a promising technique based on targeted fluorescent imaging and PDT. Photofrin and aminolevulinic acid and its ester derivatives are the main compounds used in clinical trials although newer photosensitizers (PSs) and delivery tools are being evaluated. This Special Issue on Cancers includes original articles on photochemical mechanisms, new PSs and delivery tools, cellular and tissue targets, cellular response (cell death or survival), vascular damage and immune response. We hope all the articles published in this Special Issue can help to improve this therapeutic cancer modality. 

## Research Snippets

Efficacy of 5-Aminolevulinic Acid (ALA) in Photodynamic Detection and Photodynamic Therapy in Veterinary Medicine: Osaki et al. provided an excellent summary of the use of ALA for the photodiagnosis and therapy of tumors in dogs and cats [1]. Small tumors disappeared with ALA–PDT and the photodiagnosis helped to predict this response. Regarding the mechanism, for the first time, they found a correlation between PpIX accumulation in canine carcinoma cells and the ferrochelatase mRNA expression. 

In-Vivo Optical Monitoring of the Efficacy of Epidermal Growth Factor Receptor Targeted Photodynamic Therapy: The Effect of Fluence Rate: Peng et al. showed how targeted PDT can improve PDT since it achieves significantly better tumor response, reducing the damage to surrounding healthy tissue and skin photosensitization [2]. They studied the efficacy of epidermal growth factor receptor (EGFR) targeted PDT using cetuximab bound to the phthalocyanine dye IRDye700DX as PS. According to the results, the treatment outcome strongly depends on the fluence rate used and the formation of singlet oxygen. Data showed that a low fluence rate illumination contributes to a higher PDT response and indicated that the effectiveness of targeted PDT is, like PDT, dependent on the generation of singlet oxygen and thus the availability of intracellular oxygen. 

Selective Killing of Activated T Cells by 5-Aminolevulinic Acid Mediated Photodynamic Effect: Potential Improvement of Extracorporeal Photopheresis (ECP): Darvekar et al. evaluated the ability of 5- ALA, a precursor of protoporphyrin IX in combination with blue light to targeting activated human blood T cells ex vivo as an alternative to the ECP. ECP was applied by using 8-methoxypsoralen (8-MOP) and ultraviolet-A (UV-A) light to treat cutaneous T-cell lymphoma [3]. They described that ALA-induced PpIX production took place in activated CD3+, CD4+CD25+, and CD8+ T cell populations with their subsequent killing after blue light exposure, whereas T cells were much less damaged by the treatment, as well as by monocyte-derived dendritic cells. They concluded that ALA-PDT kill activated T cells more selectively and efficiently than 8-MOP/UV-A, and proposed an ALA-PDT strategy for improving ECP also for the induction of immune tolerance. 

A Basic Study of Photodynamic Therapy with Glucose-Conjugated Chlorin e6 Using Mammary Carcinoma Xenografts: Osaki et al. have developed a new PS, a glucose-conjugated chlorin e6 (G-Ce6) against canine mammary carcinoma (CMC) in vitro and in vivo [4]. The development of the PS is based on the Warburg effect; tumoral cells consume high levels of glucose. They demonstrated the high efficacy of the compound in vitro and in vivo. 

The Ruthenium-Based Photosensitizer TLD1433 Inhibits Conjunctival Melanoma Cells in Zebrafish Ectopic and Orthotopic Tumour Models: Chen et al. evaluated the efficacy of PDT with TLD1433 and green light to treat conjunctival melanoma (CM) [5]. CM is an ocular cancer developed from mutated melanocytes in the conjunctiva and that can be deadly. This treatment triggered the death in CM cell lines via apoptosis and necrosis. TLD1433-mediated PDT was also proven in a zebrafish ectopic and newly-developed orthotopic CM models. Both intravenous and retro-orbital administration of the drug into the fish showed excellent anti-tumour properties with a low toxicity, so TLD1433 could be repurposed for the treatment of CM. 

Photochemically-Induced Release of Lysosomal Sequestered Sunitinib: Obstacles for Therapeutic Efficacy: Wong et al. have investigated whether photochemical internalization (PCI), a technology for cytosolic release of drugs entrapped in endosomes and lysosomes, would activate the sunitinib sequestered into lysosomes [6]. Lysosomal accumulation of sunitinib has been suggested as an underlying mechanism of resistance. Sunitinib was found to accumulate in the endo/lysosomal compartments together with the PS disulfonated tetraphenylchlorin (TPCS2a) in human colon cancer cells. The cytotoxic outcome of sunitinib-PCI was highly dependent on the treatment protocol. The mechanism of acquired sunitinib resistance in colon cancer cells is not clearly related to lysosomal sequestering sunitinib. The in vivo studies indicated that the tumor growth could be related with decreased infiltration of CD3-positive T cells. 

Cytoplasmic Increase in Hsp70 Protein: A Potential New Biomarker of Early Infiltration of Cutaneous Squamous Cell Carcinoma Arising from Actinic Keratosis: Fernández-Guarino et al. have evaluated the expression levels of three proteins, namely alpha hemoglobin and heat shock proteins 27 and 70 (Hsp27 and Hsp70, respectively) in biopsies of actinic keratosis (SCC-AK) to predict SCC infiltration [7]. They indicated that the expression level of Hsp70 protein, evaluated by immunohistochemistry and western blot, positively correlated with the level of SCC-AK dermis infiltration. The group concluded that cytoplasmic expression of Hsp70 could be a potential biomarker of early infiltration of SCC arising from AK.

The Potential of Nanobody-Targeted Photodynamic Therapy to Trigger Immune Responses: Beltran-Hernandez et al. showed the multitarget effect of nanobodies that bind to tumor cells with high affinity and selectively deliver the PS, which exerts a very specific cytotoxic effect after irradiation [8]. The novelty is that this process induces the production of pro- inflammatory cytokines, such as IL-1β and IL-6, which initiate a systemic immunological reaction. This means that a local selective therapy can reach a systemic effect, which could be beneficial in the fight against the oncology disease. 

Role of Polymer Micelles in the Delivery of Photodynamic Therapy Agent to Liposomes and Cells: Gibot et al. indicated that the efficiency of PDT can be reduced because of the hydrophobic properties of PSs [9]. In this regard, the encapsulation of PS inside nanovectors can improve the pharmacokinetic properties of PSs and thus the efficacy of the treatment. The work focused on the delivery of Pheophorbide a (Pheo) from polymeric micelles based on poly (ethylene oxide block-poly(ε-caprolactone) (PEO-PCL) and poly (ethylene oxide-b-styrene) (PEO-PS). The cellular uptake of Pheo and the efficacy of PDT were significantly increased when PS was administered as PEO-PCL or PEO-PS, confirming the benefits of using copolymer micelles. 

CD44 Targeting Mediated by Polymeric Nanoparticles and Combination of Chlorine TPCS2a-PDT and Docetaxel-Chemotherapy for Efficient Killing of Breast Differentiated and Stem Cancer Cells In Vitro: Gaio et al. have focused their work on cancer stem cells (CSCs), which are present in tumors [10]. Although only representing a small fraction, they are highly tumorigenic and, because of the difficulty in removing them, also favour the development of resistance to conventional chemotherapies. The improved therapeutic efficacy of combining PDT, chemotherapy and nanometric delivery systems has been recently reported by several studies. In this work, the combination of docetaxel (DTX)-chemotherapy and meso-tetraphenyl chlorine disulfonate (TPCS2a)-PDT using hyaluronic acid (HA)-covered nanoparticles (HA-NPs) was used for the in vitro treatment of breast cancer cells. Results showed that this combined treatment had superior efficacy over monotherapies because of the targeting and killing of CSCs. 

Sensitive Photodynamic Detection of Adult T-cell Leukemia/Lymphoma and Specific Leukemic Cell Death Induced by Photodynamic Therapy: Current Status in Hematopoietic Malignancies: Oka et al. demonstrated in this article that ALA-PDT is very effective in selectively eliminating in vitro Adult T-cell Leukemia/Lymphoma (ATL) cells while preserving the normal ones [11]. These findings support the use of orally administered 5-ALA, and then visible light exposure of ATL cells and normal lymphocytes in peripheral blood using the extracorporeal circulation system. This system can not only help to treat different types of leukemia with less toxicity than usual chemotherapy, but also metastatic cells can be targeted. 

Metformin as an Adjuvant to Photodynamic Therapy in Resistant Basal Cell Carcinoma Cells: Mascaraque et al. showed evidence, in vitro and in vivo in mice, that metformin improves the cytotoxic effect of MAL-PDT either in PDT sensitive or resistant cells [12]. This effect was not related with an increase in PpIX accumulation but related to the activation of pAMPK and the suppression of the mTOR pathway. The fact that the aerobic glycolysis metabolism predominates in resistant BCC cells supports the use of metformin to overcome BCC resistance to MAL-PDT. 

Comparison of Cellular Death Pathways after mTHPC-mediated Photodynamic Therapy (PDT) in Five Human Cancer Cell Lines: Lange et al. showed how Temoporfin (mTHPC) can exert phototoxic effects in different ways depending on the treated cancer cell [13]. Regarding the cell death mechanism, whereas the loss of mitochondrial membrane and an increased generation of ROS is observed in all lines, lipid peroxidation seemed to play a minor role. At low-doses, PDT autophagy can impair apoptosis, whereas at higher doses both of them are associated. Necrosis seemed not to play a relevant role. After studying these mechanisms in different types of cancer cells (lung carcinoma, oral squamous cell carcinoma, esophageal squamous cell carcinoma, urinary bladder transitional cell carcinoma and cervix adeno carcinoma), they concluded that the photodynamic dose must be adjusted for every cellular type. Therefore, it is probably that different clinical protocols are needed for the use of mTHPC to treat different types of cancers.

Selective Targeting of Cancer Stem Cells (CSCs) Based on Photodynamic Therapy (PDT) Penetration Depth Inhibits Colon Polyp Formation in Mice: Kim et al. indicated that targeting cancer stem cells (CSCs), without damaging normal stem cells, could contribute to the development of novel cancer therapies [14]. Therefore, since cells expressing leucine-rich repeat-containing G-protein coupled receptor 5 (Lgr5) constitute a cancer-causing population in the colon, targeting of Lgr5+ cells is expected to mitigate this type of cancer. They have used a modified PDT technique involving cellular radiative transfer between green fluorescent protein (GFP)-expressing cells and a rose bengal PS. They observed that Lgr5+ CSCs were specifically eradicated in situ, when localized based on the depth from the colon lumen and, therefore, may be feasible for prevention of colon cancer in high-risk populations.

Photochemical Internalization: Light Paves Way for New Cancer Chemotherapies and Vaccines: Šoši´c et al. performed a review on the state of the art in photochemical internalization (PCI), a technology employed for the cytosolic release of chemotherapeutic agents or antigens [15]. Molecules such as chemicals, proteins, DNA or RNA are internalized by cells via endocytosis of phagocytosis. Several published studies highlight the potential of PCI to enhance the treatment efficacy by releasing chemotherapeutics to the cytosol of tumor cells. Thus, PCI enhances the cytotoxicity of bleomycin (Clinical Phase I) or mediates MHC-I antigen presentation. In addition, they also indicate that a first clinical Phase I trial with the PS TPCS2a combined with human papilloma virus antigen (HPV) has been recently completed. Hence, PCI has a therapeutic potential to improve the effectiveness of some cancer therapies and help the immune system via tumor-antigens. 
